# Advances in Atomic Gyroscopes: A View from Inertial Navigation Applications

**DOI:** 10.3390/s120506331

**Published:** 2012-05-11

**Authors:** JianCheng Fang, Jie Qin

**Affiliations:** Science and Technology on Inertial Laboratory, Beihang University, Beijing 100191, China; E-Mail: fangjiancheng@buaa.edu.cn

**Keywords:** atomic gyroscope, atomic interferometer, atomic spin, cold atom, guided atom, SERF, comagnetometer

## Abstract

With the rapid development of modern physics, atomic gyroscopes have been demonstrated in recent years. There are two types of atomic gyroscope. The Atomic Interferometer Gyroscope (AIG), which utilizes the atomic interferometer to sense rotation, is an ultra-high precision gyroscope; and the Atomic Spin Gyroscope (ASG), which utilizes atomic spin to sense rotation, features high precision, compact size and the possibility to make a chip-scale one. Recent developments in the atomic gyroscope field have created new ways to obtain high precision gyroscopes which were previously unavailable with mechanical or optical gyroscopes, but there are still lots of problems that need to be overcome to meet the requirements of inertial navigation systems. This paper reviews the basic principles of AIG and ASG, introduces the recent progress in this area, focusing on discussing their technical difficulties for inertial navigation applications, and suggests methods for developing high performance atomic gyroscopes in the near future.

## 1. Introduction

Gyroscopes are one of the key sensors for Inertial Navigation Systems (INSs), and are used widely for underwater robots, cars, airplanes, satellites, *etc.* [1–3]. The Global Position System (GPS) can provide high accurate position information and is used everywhere in our daily life, but GPS cannot be used for underwater and indoor navigation. Meanwhile, GPS signals are easily disturbed, so GPS based navigation systems cannot work alone in some situations such as navigation for airplanes [4,5]. INS is based on Newton's law. To give a position, accelerations in three axes must be integrated twice while gyroscopes provide the directions to which the accelerometers are pointing. The principle of INS is simple, but position errors are accumulated rapidly due to the errors of the accelerometers and gyroscopes. With the development of modern inertial technology, high performance accelerometers are available. However, from the mechanical gyroscope to the optical gyroscope, the improvements of those gyroscopes have been very slow in the past 10 years. The best mechanical gyroscope is still in the 10^−6^°/h level, while the best optical gyroscope is in the 10^−4^°/h level [6]. For long time inertial navigation applications such as underwater robots in deep sea, the limited precision of gyroscopes has been the main problem to increase the performance of INS [7–9].

In recent years, with the rapid development of modern physics such as the progress on quantum mechanics, atomic manipulation and modern optics, especially the Nobel Prize for Physics in 1997, 2001 and 2005, the atomic interferometers using these atomic manipulation technologies has reached ultra-high sensitivity and has been used widely [10]. The Atomic Interferometer Gyroscope (AIG), which has been demonstrated in recent years, is one of the most important applications of atomic interferometers. The AIG has ultra-high sensitivity of rotation, and is believed to be the development trend for the ultra-high precision strategic grade gyroscopes in future. Meanwhile, with the deeper understanding of the atomic spin, devices based on atomic spin have been ranked as the most sensitive sensors or powerful tools for wide applications in the World. For example, the atomic clock which utilizes atomic hyperfine structure, has become the World's most accurate time standard [11]; the nuclear magnetic resonance (NMR) analyzer, which utilizes NMR phenomena of atomic spin, has become one of the most powerful tools in modern medicine [12]; and the Atomic Spin Gyroscope (ASG) is also an important sensitive atomic spin sensor for high performance inertial navigation applications, which is believed to be the development trend for the high precision and compact gyroscopes in future. In a word, with the development of modern atomic physics, AIG and ASG have been designed and demonstrated that they will be the next generation gyroscopes for future high precision inertial navigation applications [13,14]. Since AIG and ASG are based on different principles, they will be introduced and analyzed separately.

## 2. Atomic Interferometer Gyroscope

**Figure 1.**
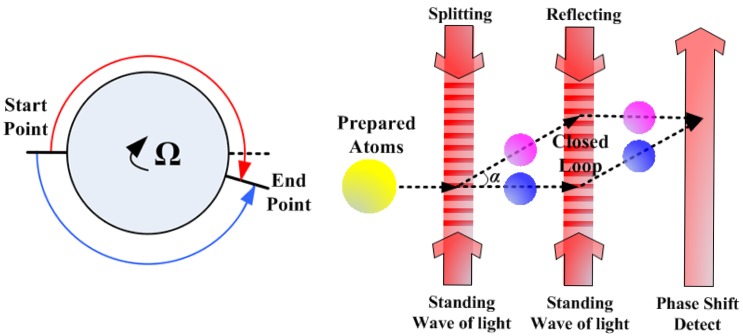
Principle of AIG.

The atom, like the photon, has the property of wave-particle duality. Like with the Sagnac effect in the optical gyroscope, the atomic interferometer can work as a gyroscope. The principle of AIG to sense rotation is shown in Figure 1. The prepared atoms are split in the start point and merged in the end point to form a closed loop, and there will be a phase shift at the end point in the presence of rotation. The fundamental principle of AIG is much like that of the optical gyroscope, but the structure of AIG is different from the optical gyroscope because the “splitter”, “mirror” and “detector” are different. As can be seen in Figure 1, for constructing an AIG, lots of atoms must be prepared first such as to be concentrated in phase space and to be selected in proper atomic states, and then, the atoms are manipulated by the standing waves of light as the gratings which are used to “split” and “reflect” atoms into a coherent supposition of momentum state [10]. The atom motion under these light gratings can form a closed loop. Finally, the laser is used to detect the phase shift of the combined atoms to tell the rotation rate.

The phase shift of AIG due to rotation is:

$$\delta \Phi_{\text{atom}}=\frac{4\pi m}{h}\Omega \cdot \text{A}$$

where *m* is the mass of the atom, *h* is the plank constant, ***Ω*** is the rotation rate vector and ***A*** is the closed loop area vector. The phase shift of optical gyroscope due to rotation is:

$$\delta \Phi_{\text{light}}=\frac{4\pi }{\lambda c}\Omega \cdot \text{A}$$

where *λ* is the wavelength of light and *c* is the speed of light. To compare the sensitivity between AIG and optical gyroscope, we have [15]:

$$\frac{\delta \Phi_{\text{atom}}}{\delta \Phi_{\text{light}}}=\frac{m}{h/(\lambda c)}>10^{10}$$

So, AIG is more sensitive to rotation by a factor of 10^10^ than optical gyroscope if they have the same closed loop area, as the mass of the atom is much larger than the relative mass of photon (*h*/*λc*).

The theoretical sensitivity of AIG is ultra-high, but the demonstrated AIG prototype in 2006 only reached 6.8 × 10^−5^°/h, which was far from its theoretical precision [16]. When comparing with optical gyroscopes, the limited performance of this AIG prototype is mainly due to the small area of the closed loop. The area in this AIG was only 22 mm^2^, which was several orders smaller than that of modern optical gyroscopes. This was because the atoms moved around the closed loop only once, and it was difficult to manipulate the atoms to move around the closed loop several times as the optical gyroscope does. On the other hand, the “splitter” and “mirror” were not effective, whereas only two photon recoil momentum were transferred to the atom motion so that the angle *α* (shown in Figure 1) between the split atoms and the incident atoms was small, which limited the atoms from forming a large closed loop, even when the atoms moved around the closed loop once [16].

The limited closed loop area of the available AIG is one of the problems to realize a high performance AIG, and to apply AIG for inertial navigation applications has other difficulties. To build an inertial measurement unit for inertial navigation, three AIG must be used to sense the attitude change of the carrier in three orthogonal directions, so the AIG not only needs to measure the rotation in the gravity direction, but also needs to measure the rotation in the horizontal direction. However, the AIG is based on the atomic interferometer, which can sense acceleration at the same time, especially the gravitational acceleration [17,18]. The most important thing is that the typically phase shift induced by the acceleration is 5–6 orders larger than induced by the rotation [19]. For a typical three pulse atomic interferometer, the total phase shift is [20,21]:

$$\delta \Phi_{\text{atom}}=\frac{4\pi m}{h}\Omega \cdot \text{A}+\text{k}\cdot \text{g}T^{2}-\text{k}\cdot (\Omega \times \text{g})T^{3}$$

where ***k*** is the effective light propagation vector, ***g*** is the gravitational acceleration and *T* is the atom propagation time. In the presence of gravity, the phase shift mainly includes three items, which are rotation rate, gravitational acceleration and the cross product of rotation rate and gravitational acceleration. Because of the phase shift induced by gravitational acceleration is relative larger than the rotation rate in the presence of tiny rotations, the items of gravitational acceleration and the cross product of rotation rate and gravitational acceleration are strong while the item of rotation rate is faint, which indicates that the signal to noise ratio (SNR) is low for the AIG. To improve the SNR of the AIG, traditional atomic interferometry methods such as the balanced three light pulses [22,23] and the four light pulses [19,24,25] can be used to cancel the item of gravitational acceleration in the total phase shift. However, the cross product of rotation rate and gravitational acceleration cannot be overcome by these methods, which means that the gravitational acceleration should be measured accurately enough or kept strictly constant both in magnitude and direction, otherwise the gravitational acceleration is the noise which will decrease the precision of AIG for long time navigation applications. That is why the current available AIG can measure the rotation in the gravity direction very accurately (the cross product of rotation rate and gravitational acceleration is zero at this situation) when the AIG is in a laboratory environment, but the sensitivity of the rotation in the horizontal direction is decreased significantly, even it is equipped with a high performance vibration isolation system [26]. Gravitational acceleration is only one kind of acceleration, and other accelerations can also have a significant influence on the AIG since the changed accelerations affect the sensitivity and scale factor of AIG via the cross product of rotation rate and acceleration. Thus, AIG for inertial navigation applications must isolate the influence of acceleration effectively.

The volume of the AIG is a very important parameter when the AIG is considered for inertial navigation applications. The volume of the AIG with 6.8 × 10^−5^°/h [16], was above 2 m in one direction, in that the atomic sources used in that system were hot atoms that had high velocity. If the atomic sources are changed to cold atoms, the side length of the closed loop will be shorter than that of the hot atoms, but for the same atom propagation time, the area of the closed loop decreases with cold atoms, which will decrease the sensitivity of the AIG. To maintain the same sensitivity, one way is to increase the cold atom propagation time so as to increase the area of the closed loop, but the bandwidth of the AIG decreases at the same time, so, the limited bandwidth of the AIG with cold atoms is still a problem that must be overcome for future applications, and the balance between the sensitivity and the bandwidth must be considered carefully for different inertial navigation application fields [27,28].

AIG with guided atoms is a new atomic manipulation method developed in recent years [29–32]. The atoms, guided by a magnetic potential field or light potential field, can be suspended in these fields to isolate the influence of the gravity and other accelerations as well. Moreover, the guided atom AIG is able to guide the atoms to move around the closed loop several times to increase the closed loop area, which will not only increase the sensitivity of the AIG but also make the whole system of compact size. However, when implementing the guided atom technology with atomic interferometry methods, the traditional internal state atomic interferometry method (where the internal state of the atom is changed) such as Raman pulse [10] cannot be integrated with guided atoms because the different atomic states are difficult to guide in the same potential field; on the other hand, traditional single state atomic interferometry method (where the internal state of the atom remains unchanged) such as Talbot-Lau pulse, can be used with guided atoms, but the sensitivity with this method is relative lower [10,30,31]. Thus, for the guided atom approach, the AIG technique needs a new atomic interferometry method.

Numerous scientists and engineers have contributed greatly to promote AIG, and several research groups have demonstrated gyroscope effects with their AIG research platforms. They will be introduced in the following paragraphs.

The research group at Stanford University demonstrated the World's first AIG with a sensitivity of 1.1 × 10^−6^°/s/Hz^1/2^ in 1997 [33], then improved the sensitivity to 3.4 × 10^−8^°/s/Hz^1/2^ in 2000 [22], and obtained a bias stability of 6.8 × 10^−5^°/h in 2006 [16]. However, these AIG used hot atoms as atomic sources, and the volume of the whole system was still large. In order to demonstrate a compact system for inertial navigation applications, they designed an AIG with cold atoms and realized a angular random walk (ARW) of 2.3 × 10^−3^°/h^1/2^ in 2008 while keeping the whole volume under 1 m^3^ [21]. To achieve a high performance AIG, they also proposed to use optical lattices to guide and split the atoms [32]. Recently, by employing a novel π/2-π-π-π/2 pulse sequence, they improved the ARW of the AIG with cold atoms to 2.95 × 10^−4^°/h^1/2^, and a rotation rate in excess of 5.73°/s can be measured [34]. To further improve the sensitivity of AIG, they wanted to form a large area AIG, and demonstrated a sequential Bragg large momentum transfer beam splitter, which can transfer 102 photon recoil momentum to the atom motion so that the angle *α* can be increased significantly [35].

The research group at Harvard University focused on the AIG with magnetic guided atoms. In 2007, they demonstrated the World's first AIG with guided atoms [30]. This AIG was able to guide the cold atoms to move around the closed loop twice, and resolved 10 times the Earth's rotation rate per interferometry cycle. In recent years, they have focused on the delta-kicked rotor to improve the coherence of the atoms during the interferometry cycle to increase the times of the guided atoms which moves around the loop [36,37], and they designed a new macroscopic magnetic guide based on nonsymmetrical geometries to decrease the guided potential field noise to improve the guided atomic interferometer performance [38].

The research group at Laboratoire National de Métrologie et d'Essais-Système de Références Temps-Espace demonstrated the World's first AIG with cold atoms in 2006 [26]. This AIG had a sensitivity of 8 × 10^−6^°/s/Hz^1/2^ in the gravity direction and a sensitivity of 1 × 10^−4^°/s/Hz^1/2^ in the horizontal direction. To investigate the various noises which establish the sensitivity limitations of the demonstrated AIG, they analyzed the sensitivity function in the AIG and pointed out that the Raman phase and residual vibrations were the main noises of the AIG, and proposed methods to decrease these noises [39]. In 2009, they achieved a new Raman transition with a symmetric momentum-space splitting of four photon recoil momentum, and increased the area of the closed loop by a factor of two [40]. Then, an improved atomic interferometer gravimeter was demonstrated based on this beam splitter [41]. To increase the interrogation time and decrease the vibration noise, they operated their atomic interferometer in the microgravity environment of an airplane in free fall, and achieved significant improvements of the atomic interferometer accelerometer [42,43] which will hopefully improve the AIG performance as well. In recent years, they cooperated with other scientists to propose a Space Atom Interferometer project funded by European Space Agency, and wanted to place the atomic interferometer in space to demonstrate its possibility for future space missions [44,45].

The research group at Leibniz Universität Hannover has focused on designing a compact and high resolution AIG. They have designed a compact ^87^Rb atomic source for AIG based on the two stage magneto optical trap (MOT) with a two dimensional MOT and a three dimensional MOT, and have realized a high atomic flux with 10^10^ atoms/s [46]. Based on this high performance atomic source, they demonstrated an AIG with a dual atom interferometer to cancel acceleration, and a sensitivity of 1.1 × 10^−2^°/s/Hz^1/2^ within interrogation time of up to 4 ms has been achieved [47].

In a word, although there are lots of difficulties that need to be overcome before the AIG for inertial navigation applications can be a reality, the future of the AIG technology is still bright due to its theoretical ultra-high sensitivity and the development of atomic manipulation technology. As described in the analysis above, the approach of “cold atom + guided atom” maybe a feasible way to develop AIG in the future for inertial navigation applications which can keep the AIG in a compact size, improve its sensitivity and isolate the accelerations.

## 3. Atomic Spin Gyroscope

**Figure 2.**
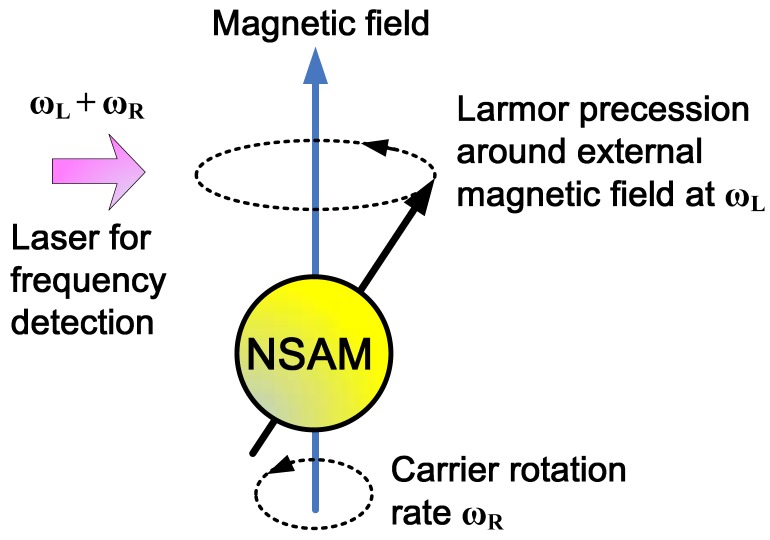
Principle of ASG based on NMR.

For an ensemble of atoms, the atomic spin is able to keep pointing at its original direction in the inertial coordinate system, which is similar to the mechanical rotor in the traditional mechanical gyroscope, so the atomic spin can be utilized to sense rotation as well. Since the 1960s, scientists and engineers have developed a new gyroscope, which uses nuclear spin angular momentum (NSAM) for rotation measurement [48–50]. Since NMR technology was used for signal detection, the gyroscope was also called NMR gyroscope. Singer and Litton had separately developed the prototype of ASG based on NMR, whose accuracy both reached the navigation grade [51,52]. The principle of this type of ASG is shown in Figure 2.

The NSAM is able to keep pointing in its original direction in the inertial coordinate system. Since NSAM has magnetic moment as well, when there is an external magnetic field, the NSAM will precess around the external magnetic field with Larmor precession frequency (LPF) [53,54]. A detection laser which fixed in the carrier coordinate system is used to detect the precession frequency of the NSAM. When the carrier rotates, the NSAM keeps its original LPF in the inertia coordinate system, but the precession frequency detected by the laser is the sum of the LPF of NSAM and the carrier rotation rate. As the LPF of NSAM is determined by the gyromagnetic ratio of the nucleon and the strength of the magnetic field, the LPF can be calculated theoretically when the gyromagnetic ratio and the magnetic field are known [55], so the carrier rotation rate can be obtained if the LPF is deducted from the frequency detected by the laser.

Replacing the mechanical rotor with atomic spin, the ASG based on NMR does not have any mechanical friction and moving parts when compared to traditional mechanical gyroscopes. The ASG has a lot of prominent features such as high precision, compact size and high impact resistance, which became a hot research field in many countries at that time. However, to accurately measure the rotation rate, the magnetic field must be controlled precisely so that the carrier rotation rate can be effectively distinguished from the detected precession frequency. To generate a stable and homogeneous magnetic field environment, superconducting coils and superconducting shield had been used [56,57], but with superconducting technology, the ASG is expensive and the whole volume of the ASG is large. Another way to overcome the unstable magnetic field problem is by using two nucleon species [58]. Since these two species are in the same magnetic field and same rotation frame, with the precession frequency of these nucleons are detected separately, the rotation rate can be obtained even with unknown magnetic fields. However, in a real system, the magnetic fields experienced by these two nucleon species are different, such as polarization dependent frequency shifts from collision between alkali metal atoms and noble gas atoms [59,60]. In addition to a magnetic field, an inhomogeneous electronic field also affects the precession frequency of NSAM [61–63], which results in a significant drift of the ASG. Therefore, because of these limitations, the ASG based on NMR only achieved navigation grade precision at that time. When comparing with the optical gyroscopes developed at the same time, lots of institutes and companies gradually quit doing research on the NMR-based ASG technology.

However, with the development of microfabrication technology, the ASG based on NMR has received new attentions recently [64]. The structure of this ASG is simple, allowing it to be manufactured with microfabrication processes. Due to its high sensitivity even with a small volume, the chip-scale ASG is a new research direction for navigation grade micro gyroscopes. The research group at National Institute of Standards and Technology designed a chip-scale ASG based on NMR with a diverging beam to form both the pump and probe light [65,66]. The research group at Northrop Grumman did a lot of great work to develop a chip-scale ASG based on NMR in recent years, from general structure and method design [60,67,68] to micro cell fabrication [69,70], and many novel ideas have been proposed. Since ASG based on NMR has been developed now for several decades, with the technology developed for chip-scale atomic sensor such as atomic clock [71] and atomic magnetometer [72,73], the chip-scale ASG based on NMR is expected to reach navigation grade precision in the near future, which was previously difficult to achieve with the existent MEMS gyroscopes.

**Figure 3.**
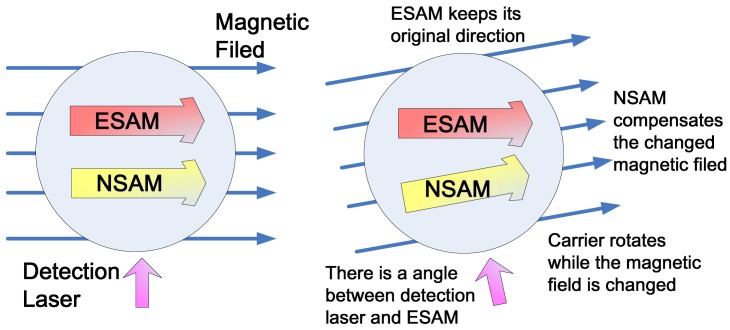
Principle of ASG based on comagnetometer.

In 2005, Princeton University researchers discovered a new type of ASG [74,75], which used a Ø25mm sphere cell to achieve a ARW of 2 × 10^−3^°/h^1/2^ and a bias stability of 4 × 10^−2^°/h, but the theoretical performance of this ASG is orders higher. This type of ASG, which utilized both electron spin angular moment (ESAM) of alkali metal atoms and NSAM of noble gas atoms to form a comagnetometer structure for rotation rate measurement, is different from the ASG based on NMR mentioned above. The principle of the ASG based on comagnetometer is shown in Figure 3. The ESAM is able to keep pointing at its original direction in the inertial coordinate system. In order to avoid the Larmor precession of ESAM in the external magnetic field, noble gas atoms are added in the same cell. Under special situations, the NSAM of noble gas atoms can strongly interact with the ESAM of alkali metal atoms. The NSAM and ESAM couple together, and the NSAM has two assets in such a comagnetometer configuration. The first one is that the NSAM is able to automatically track and compensate for the changes of the external magnetic field, thus isolating the external magnetic field sensed by the ESAM. When the carrier rotates, the ESAM will remain in its original pointing direction. Since the detection laser is fixed in the carrier, the angle, which is between the ESAM and the detection laser, reflects the rotation only. In addition, the NSAM can enhance the component of ESAM that rotates into the detection laser direction as the carrier rotates so it increases the ability of ESAM to sense rotation [13].

The fundamental sensitivity of the ASG based on comagnetometer is [76]:

$$\delta \Omega =\frac{\gamma_{n}}{\gamma_{e}}\sqrt{\frac{1}{nVT_{2}t}}$$

where *γ_n_* is the nuclear gyromagnetic ratio of the noble gas atom, *γ_e_* is the electron gyromagnetic ratio of the alkali metal atom, *n* is the density of alkali metal atoms, *V* is the active volume for detected alkali metal atoms, *T_2_* is the relaxation time of the alkali metal atom and *t* is the measurement time. Since this ASG is based on the atomic magnetometer, the fundamental sensitivity of the atomic magnetometer is [77]:

$$\delta B=\frac{1}{\gamma_{e}}\sqrt{\frac{1}{nVT_{2}t}}$$

To calculate the fundamental sensitivity of the ASG based on comagnetometer, we know the the atomic magnetometer can be realized with better than 1aT/Hz^1/2^ sensitivity [77] with a measurement volume of around 150 cm^3^. If ^21^Ne is used where *γ_n_* is 2.1 × 10^7^rad/s/T, the sensitivity of the ASG based on comagnetometer is 1.2 × 10^−9^°/s/Hz^1/2^ (an equal ARW of 7.2 × 10^−8^°/h^1/2^), which indicates that this type of ASG has outstanding advantages of high sensitivity and compact size. The measurement volume around 150 cm^3^ is quite small when compared with traditional mechanical and optical gyroscopes, and the performance of the ASG based on comagnetometer can be further improved with increased measurement volume.

The ASG based on comagnetometer does not need to precisely control the magnetic field when compared to the ASG based on NMR. The direction change of the ESAM is only due to the rotation, which is different from the ASG based on NMR. Moreover, the sense direction of the ASG based on comagnetometer is in the vertical direction of the plane formed by the pump and probe lasers, while the sense direction of the ASG based on NMR is in the applied external magnetic field.

The demonstrated ASG based on comagnetometer used alkali metal atoms which are in the Spin-Exchange Relaxation Free (SERF) regime [78,79] to increase the fundamental sensitivity of rotation. As it can be seen in the fundamental sensitivity equation of the comagnetometer-based ASG, to increase the sensitivity of rotation, the item of n·*T_2_* should be increased. However, with the increased density of atoms, the relaxation time will normally decrease dramatically, mainly due to the increased spin exchange relaxation between atoms. When the atoms are in SERF regime, the atoms not only have the characteristics of high atomic density so as to improve SNR of the whole system, but also have longer relaxation time which can be increased by three orders when compared with atoms in the traditional optical pumping regime at the same atomic density, so the SERF enables the alkali metal atoms to achieve both high density and long relaxation times so as to produce a high performance ASG [80].

To consider the ASG based on comagnetometer for inertial navigation applications, several problems must be overcome. Firstly, the start-up time of the gyroscope must be considered. The realized ^3^He-K ASG needed several hours to spin exchange optical pumping of ^3^He [81], and then the NSAM of ^3^He can be worked to form the comagnetometer, which cannot be used in some inertial navigation situations with quick start-up requirements. To overcome this defect, one needs to change the atomic spin species used in ASG, such as the NSAM of ^129^Xe, which is easier to be spin exchange optical pumped by Cs within several minutes. However, the fundamental sensitivity of ^129^Xe-Cs ASG is lower than ^3^He-K ASG due to its shorter relaxation time, so for future inertial navigation applications, it must be a balanced choice between start-up time and sensitivity. Secondly, the NSAM played an important role to help the ESAM not be affected by the magnetic field, but the strength of the compensation field generated by NSAM is limited, and the NSAM cannot automatically compensate the magnetic field very fast. This will be a big problem when the ASG based on comagnetometer is used in strapdown INS. The ways to solve this problem maybe by increasing the strength of the NSAM and using feedback control of the NSAM and ESAM to form a closed loop ASG so as to increase the measurement range and bandwidth. Thirdly, further improvement of the ASG must be considered to compete with the available gyroscopes. At present, to further improve the sensitivity of ASG, one can increase the relaxation time of the atomic spin and use noble gas atoms which have smaller nuclear gyromagnetic ratios. Since the alkali metal atoms are already in the SERF regime, to further increase relaxation time, the surface coating of the alkali metal cell at high temperature must be solved [82,83]. On the other hand, ASG based on comagnetometer is a dual axis gyroscope by nature, so the dual axis ASG could be realized in the near future.

The ASG based on comagnetometer has been developed only in the past 7 years, but the performance of ASG is comparable to the best AIG to-date. The research group at Princeton University discovered the ASG based on comagnetometer when conducting CPT violation experiments in 2005 [75]. Since then, they built a second generation research platform integrating several new features, which mainly focused on combating long term drift [84,85]. Although they used this device for CPT violation experiment, the device essentially cannot distinguish the rotation from CPT violation. In 2010, the second generation research platform achieved 0.7 nHz energy resolution for CPT violation (an equal resolution of 2.52 × 10^−7^°/s of gyroscope), which is nearly 100 times better than the 60 nHz they reached in 2005 [86]. They did not show the test data when this device worked as a gyroscope, but the performance of this ASG should be orders better than the previous one. Since the nuclear gyromagnetic ratio of ^21^Ne is an order lower than ^3^He, the fundamental sensitivity of the ASG should be improved by a factor of 10 if ^3^He is replaced with ^21^Ne. In 2011, they used ^21^Ne-Rb-K to form an ASG and reached a 0.5 nHz energy resolution [87]. Such available techniques can increase the performance of the ASG based on comagnetometer significantly.

**Figure 4.**
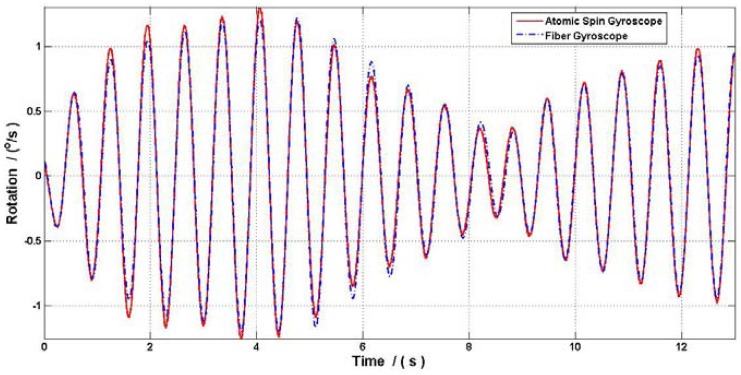
The ASG signal compare with fiber gyroscope.

**Figure 5.**
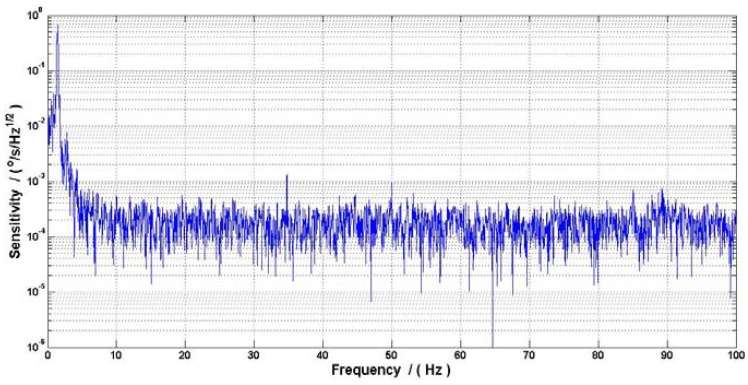
The Fourier spectrum of the ASG noise.

Our research group at Beihang University proposed a new ASG based on comagnetometer which used ^129^Xe-Cs. The relaxation time in this ASG is shorter than the ASG that used ^3^He or ^21^Ne which resulted in a lower fundamental sensitivity of rotation, but the ^129^Xe is used to decrease the start-up time. This ASG can be set up by spin exchange optical pumping between Cs and ^129^Xe for around several minutes, which is shorter than the several hours used in ^3^He-K ASG. To test the gyroscope effect, the ASG research platform was air floated and vibrated by hand with a small angle, and a high precision fiber gyroscope with 0.03°/h was used to calibrate the gyroscope signal. Figure 4 showed the ASG signal compared with the fiber gyroscope, and Figure 5 shows that the ASG reached a sensitivity of 7 × 10^−5^°/s/Hz^1/2^ (an equal ARW of 4.2 × 10^−3^°/h^1/2^) with the scale factor calibrated by the fiber gyroscope. The peak signal in Figure 5 is the resonant frequency of the ASG research platform.

Like the ASG based on NMR, the chip-scale ASG based on comagnetometer was also developed. The research group at Honeywell designed a chip-scale ASG based on its chip-scale atomic clock research experience [88], the integration methods and microfabrication processes for each elements of this ASG were designed.

In a word, the ASG based on comagnetometer has pointed a new way to obtain a highly sensitive and compact gyroscope, and with the development of microfabrication technology, the chip-scale ASG has also pointed a new way to obtain a navigation grade microgyroscope. Since several companies have been involved in the development of ASG, it seems that the ASG is ready for inertial navigation applications in the near future.

## 4. Outlook and Conclusions

Recent progress on atomic gyroscopes has demonstrated that AIG is an ultra-high precision gyroscope for strategic grade inertial navigation in the future, and ASG is a high performance gyroscope which features both high precision and compact size. There are still lots of problems that need to be overcome when considering the atomic gyroscope for inertial navigation applications, however with the technologies available in the near future, the AIG could be used in space for fundamental physics research, the ASG based on comagnetometer can be used in strategic grade gimbaled INS, and the navigation grade ASG based on NMR can be demonstrated on chip-scale size. Furthermore, with the development of new theories and technologies for atomic manipulation, the future of high performance inertial navigation applications using AIG and ASG is indeed bright.
